# Effect of HIV-1-related protein expression on cardiac and skeletal muscles from transgenic rats

**DOI:** 10.1186/1742-6405-5-8

**Published:** 2008-04-25

**Authors:** Jeffrey S Otis, Yaroslav I Ashikhmin, Lou Ann S Brown, David M Guidot

**Affiliations:** 1 Pulmonary, Allergy and Critical Care Medicine, Atlanta VA Medical Center and Emory University School of Medicine, 1670 Clairmont Road, Decatur, GA 30033, USA; 2 I.M. Sechenov Moscow Medical Academy, Moscow, Russia; 3 Department of Pediatrics, Emory University School of Medicine, 2015 Uppergate Drive, Atlanta, GA 30322, USA

## Abstract

**Background:**

Human immunodeficiency virus type 1 (HIV-1) infection and the consequent acquired immunodeficiency syndrome (AIDS) has protean manifestations, including muscle wasting and cardiomyopathy, which contribute to its high morbidity. The pathogenesis of these myopathies remains partially understood, and may include nutritional deficiencies, biochemical abnormalities, inflammation, and other mechanisms due to viral infection and replication. Growing evidence has suggested that HIV-1-related proteins expressed by the host in response to viral infection, including Tat and gp120, may also be involved in the pathophysiology of AIDS, particularly in cells or tissues that are not directly infected with HIV-1. To explore the potentially independent effects of HIV-1-related proteins on heart and skeletal muscles, we used a transgenic rat model that expresses several HIV-1-related proteins (e.g., Tat, gp120, and Nef). Outcome measures included basic heart and skeletal muscle morphology, glutathione metabolism and oxidative stress, and gene expressions of atrogin-1, muscle ring finger protein-1 (MuRF-1) and Transforming Growth Factor-β_1 _(TGFβ_1_), three factors associated with muscle catabolism.

**Results:**

Consistent with HIV-1 associated myopathies in humans, HIV-1 transgenic rats had increased relative heart masses, decreased relative masses of soleus, plantaris and gastrocnemius muscles, and decreased total and myosin heavy chain type-specific plantaris muscle fiber areas. In both tissues, the levels of cystine (Cyss), the oxidized form of the anti-oxidant cysteine (Cys), and Cyss:Cys ratios were significantly elevated, and cardiac tissue from HIV-1 transgenic rats had altered glutathione metabolism, all reflective of significant oxidative stress. In HIV-1 transgenic rat hearts, MuRF-1 gene expression was increased. Further, HIV-1-related protein expression also increased atrogin-1 (~14- and ~3-fold) and TGFβ_1 _(~5-fold and ~3-fold) in heart and plantaris muscle tissues, respectively.

**Conclusion:**

We provide compelling experimental evidence that HIV-1-related proteins can lead to significant cardiac and skeletal muscle complications independently of viral infection or replication. Our data support the concept that HIV-1-related proteins are not merely disease markers, but rather have significant biological activity that may lead to increased oxidative stress, the stimulation of redox-sensitive pathways, and altered muscle morphologies. If correct, this pathophysiological scheme suggests that the use of dietary thiol supplements could reduce skeletal and cardiac muscle dysfunction in HIV-1-infected individuals.

## Background

Although infection with the human immunodeficiency virus type 1 (HIV-1) is more commonly associated with serious derangements to the central nervous, pulmonary, and lymphatic systems, the acquired immunodeficiency syndrome (AIDS) can also produce significant cardiac and skeletal muscle dysfunction. For example, HIV-1-related cardiomyopathies may include left ventricular dysfunction, dilatation, and heart failure [1]. Further, skeletal muscle derangements due to HIV-1 infection may include polymyositis, rhabdomyolysis, tumor infiltrations, wasting syndromes, severe weakness, and fatigue [2,3].

The pathogenesis of HIV-1-associated myopathies is not fully understood, but has been attributed in part to poor nutritional states, elevated cytokine levels, oxidative stress, and other mechanisms associated with viral infection and replication [2,4,5]. Interestingly, evidence has evolved implicating HIV-1-related proteins, including gp120 and Tat, as mediators of injury even when target cells are not directly infected with HIV-1 [6-10]. For example, elevated levels of HIV-1 RNA in plasma correlate with decreased skeletal muscle amino acid metabolism and protein synthesis rates [6]. HIV-1 transcripts have also been detected in a small number of myocardial cells [7]; and the targeted expression of HIV-1 Tat in mouse hearts resulted in significant oxidative stress and severe myocardial derangements suggesting a predominant role of oxidative stress in HIV-1-related cardiomyopathies [8]. However, the influence of HIV-1-related protein-induced oxidative stress on specific redox-sensitive mechanisms in cardiac and skeletal muscle tissues remains largely unknown.

We have recently shown that two catabolic factors, atrogin-1 and Transforming Growth Factor-β_1 _(TGFβ_1_), are sensitive to oxidative stress in skeletal muscles from alcohol-fed rats [11]. Based on these observations and strong evidence that HIV-1 is also associated with increased oxidative stress [12], the aim of the current study was to determine the potential roles these redox-sensitive factors may play in HIV-1 myopathies. In addition, we analyzed the expression levels of muscle ring finger protein-1 (MuRF-1); that, like atrogin-1, is a muscle specific E3 ligase implicated in muscle atrophy [13]. Taking advantage of a non-replicative, non-infectious HIV-1 transgenic rat model [14], we show that chronic expression of HIV-1-related proteins causes significant cardiac and skeletal muscle morphological derangements including increased relative heart masses and muscle atrophy. These derangements may be due in part to increased oxidative stress, with particular alterations to glutathione metabolism, and increased expressions of atrogin-1, MuRF-1 and TGFβ_1_.

## Results

### Gross pathology of HIV-1 transgenic rats

**Figure 1:**
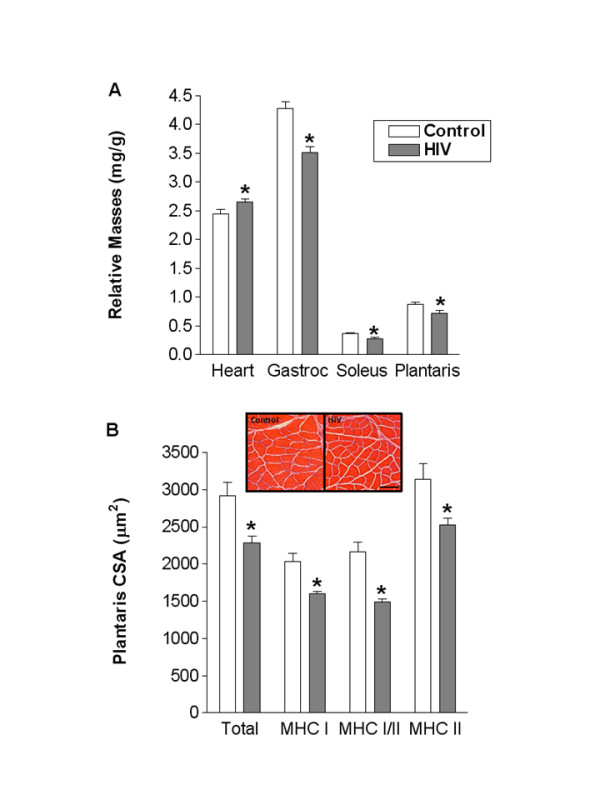
**Gross pathology of heart and plantaris muscles from HIV-1 transgenic rats**. (A) Relative heart masses from HIV-1 transgenic rats were increased compared to controls. In addition to this cardiac tissue defect, several skeletal muscles from HIV-1 transgenic rats were atrophied, including gastrocnemius (gastroc), soleus, and plantaris. (B) Specifically, the cross-sectional areas (CSA) of total and myosin heavy chain (MHC) isoform type-specific (i.e., slow, hybrid or fast MHC isoforms) from plantaris fibers were reduced in HIV-1 transgenic rats. Data in panel A represented as milligram of tissue weight divided by body mass in grams. Bar in panel B = 100 μm. *, p ≤ 0.05 vs. control.

Preliminary data showed that heart and skeletal muscle tissues from young HIV-1 transgenic rats (e.g., 2–4 months) do not exhibit any HIV-1 related defects in morphology. These initial observations are in agreement with those of Reid and colleagues that suggested HIV-1 associated complications in these transgenic rats manifest between 5–9 months of age [14]. We now show that 7 month old HIV-1 transgenic rats also have significantly larger relative heart masses, atrophied gastrocnemius, soleus and plantaris muscles (Fig. 1A), and decreased total and MHC-specific plantaris fiber areas (Fig. 1B).

### Oxidative stress in muscle tissues from HIV-1 transgenic rats

**Figure 2:**
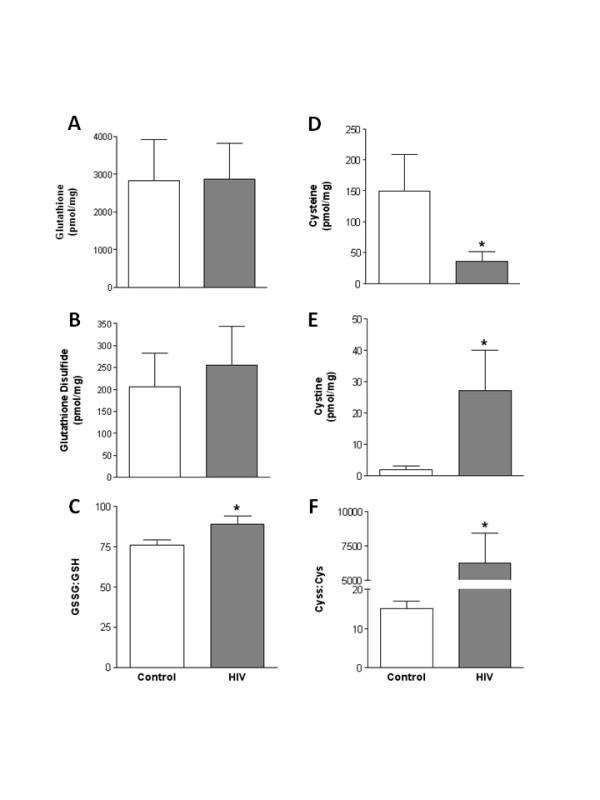
**GSH and Cys pools in heart tissues from HIV-1 transgenic rats**. High performance liquid chromatography was performed on heart tissues to detect levels of the thiol pairs GSH and GSSG, and Cys and Cyss. HIV-1-related protein expression had no effect on GSH or GSSG levels (A and B), but did increase the overall oxidative state of the GSH pool (C). In contrast, Cys levels were reduced and Cyss levels were elevated in heart tissues from HIV-1 transgenic rats compared to controls (D and E, respectively). Therefore, the Cyss:Cys ratio, a marker of the overall oxidative state of the Cys pool, was significantly increased in HIV-1 transgenic rat hearts (F). *, p ≤ 0.05 vs. control.

**Figure 3:**
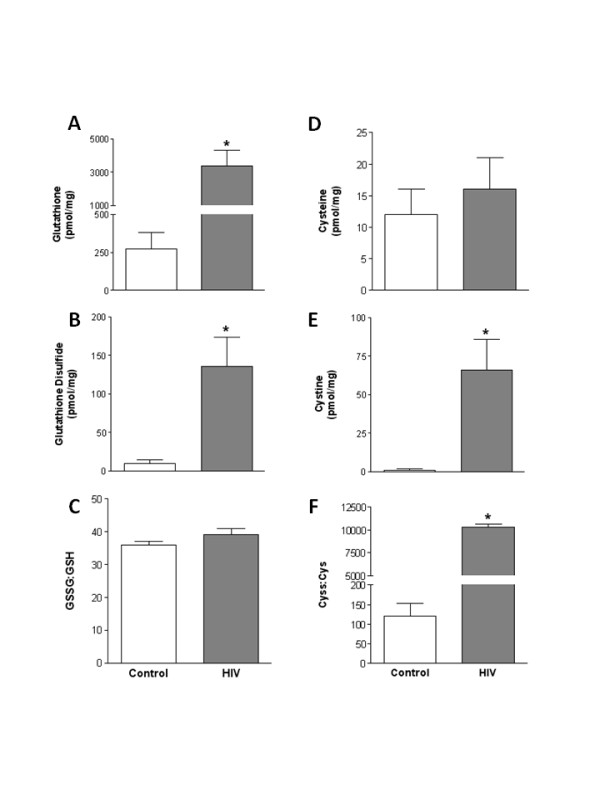
**GSH and Cys pools in plantaris muscles from HIV-1 transgenic rats**. High performance liquid chromatography was performed on plantaris muscles to detect levels of the thiol pairs GSH and GSSG, and Cys and Cyss. HIV-1-related protein expression increased the levels of GSSG (B) and Cyss (E) compared to controls. Surprisingly, GSH levels were markedly increased in plantaris muscles from HIV-1 transgenic rats (A), which served to normalize the overall oxidative state of the GSH pool (C). In contrast, the overall oxidative state of the Cys pool was significantly increased in HIV-1 transgenic rat plantaris muscles (F). *, p ≤ 0.05 vs. control.

HIV-1 infection is associated with increased oxidative stress [5]. Therefore, we next identified the effect of HIV-1-related protein expression on the glutathione (GSH) anti-oxidant system in heart and plantaris muscles. In heart tissues, no effects of the transgene were evident on the levels of GSH or glutathione disulfide (GSSG) (Fig. 2A and 2B, respectively). However, the GSSG:GSH ratio, a marker of the oxidative state of the GSH pool, was significantly elevated in heart tissues from HIV-1 transgenic rats (Fig. 2C) suggesting increased oxidative stress to this thiol pool. Heart tissues from HIV-1 transgenic rats also had significantly lower levels of cysteine (Cys), higher levels of cystine (Cyss), and an elevated Cyss:Cys ratio (Fig. 2D–F, respectively). Interestingly, both GSH and GSSG level were increased in plantaris muscles from HIV-1transgenic rats compared to controls (Fig. 3A and 3B, respectively). However, there was no difference in the GSSG:GSH ratio between these groups suggesting that the GSH pool was largely unaffected by the products of the transgene (Fig. 3C). In contrast, plantaris muscles from HIV-1 transgenic rats had increased Cyss levels and an increased Cyss:Cys ratio suggesting significant oxidative stress to this thiol pool (Fig. 3E and 3F, respectively).

### Atrogin-1, Muscle ring finger protein-1 (MuRF-1), and Transforming Growth Factor-β_1 _(TGFβ_1_) expressions

**Figure 4:**
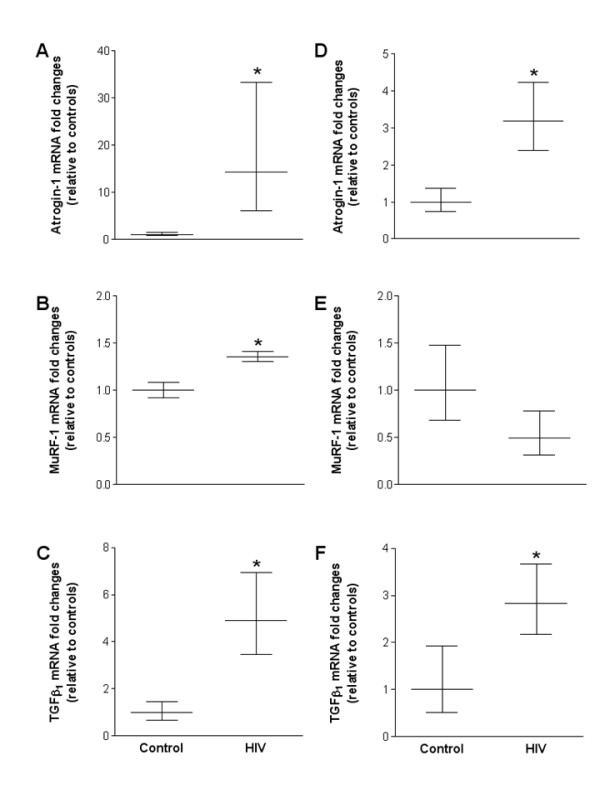
**Atrogin-1, MuRF-1 and TGFβ_1 _mRNA expression patterns in cardiac and plantaris tissues from HIV-1 transgenic rats**. Gene expression levels of several catabolic factors including, atrogin-1, MuRF-1 and TGFβ_1_, were markedly increased in HIV-1 transgenic rat heart tissues compared to controls (A-C, respectively). Similarly, mRNA expression levels of atrogin-1 and TGFβ_1 _were increased in plantaris muscles from HIV-1 transgenic rats compared to controls (D and F, respectively), however, no changes were detected in the levels of MuRF-1 (E). *, p ≤ 0.05 vs. control.

Using a model of chronic alcohol ingestion to induce oxidative stress in skeletal muscle, we have recently identified atrogin-1 and TGFβ_1 _as redox-sensitive catabolic factors [11]. However, whether or not these factors or MuRF-1 were also sensitive to HIV-1-related protein-induced oxidative stress was unknown. Atrogin-1 levels increased ~14- and ~3-fold in heart and plantaris muscles from HIV-1 transgenic rats, respectively (Figures 4A and 4D). Interestingly, MuRF-1 mRNA levels were only increased in HIV-1 transgenic rat hearts (Figure 4B). Gene levels of TGFβ_1 _were increased ~5- and ~3-fold in heart and plantaris muscles from HIV-1 transgenic rats, respectively (Figures 4C and 4F).

## Discussion

In this study, we examined two muscle types from HIV-1 transgenic rats and report significant morphological derangements, including increased relative heart weights, decreased relative masses of the plantaris, soleus and gastrocnemius, and plantaris fiber atrophy. In both tissue types, these effects were associated with increased oxidative stress, as reflected by alterations in the cysteine and glutathione redox balances. In parallel, we determined that HIV-1-related protein expression alone, in complete absence of viral replication and infection, is sufficient to induce atrogin-1 and TGFβ_1 _gene expressions, two factors strongly implicated in muscle catabolism. We also showed that the E3 ubiquitin ligase, MuRF-1, was significantly upregulated in HIV-1 transgenic rat hearts. Together, these data suggest an important and previously unrecognized relationship in HIV-1 myopathies between the bioactivity of HIV-related proteins and oxidative stress-mediated signaling events. These findings may also suggest that dietary anti-oxidant therapy with thiols such as S-adenosyl-methionine, N-acetylcysteine, or procysteine may reduce the influences of oxidative stress and/or redox-sensitive signaling pathways in HIV-1-infected individuals.

HIV-1 infection leads to impaired antigen-specific T cell proliferation and heightened susceptibility to apoptosis. Similarly, HIV-1 transgenic rats, despite the absence of characteristic viral disease progression, have an absolute reduction in CD4+, a reduced number of IFN-gamma-producing CD8+ T cells, and an increased susceptibility of T cells to activation-induced apoptosis [15]. Likewise, HIV-1 transgenic rats develop many clinical manifestations by 5–9 months of age that resemble AIDS, including neurological abnormalities, mild interstitial pneumonia, and endocarditis [14]. We now show that HIV-1 transgenic rats also have increased relative heart weights and significant skeletal muscle atrophy – consistent with cardiac and skeletal myopathies seen in individuals with AIDS. For example, reports have suggested extensive left ventricular hypertrophy and elevated heart weights in HIV-1-infected children [16]. Further, HIV-1-infected individuals may present with significant loss of lean body mass, skeletal muscle wasting, and concomitant reductions in functional capacity [2,3,17]. In this experimental study, plantaris fiber atrophy was apparent in both fast and slow myosin heavy chain (MHC) fiber types in HIV-1 transgenic rats. Further, soleus and gastrocnemius muscles were atrophied in these transgenic rats (data not shown) suggesting that HIV-1-related protein expression induces systemic atrophy that is neither fiber-type nor muscle-type specific. Interestingly, our data are in contrast to a recent report that showed type II fiber-specific atrophy in extensor digitorum longus (EDL) and gastrocnemius muscles with preserved type I fiber area in soleus muscles from a transgenic mouse model of HIV-1 (i.e., "Tg26") [17]. We did not distinguish between the fast subtypes of MHC isoforms found in rats (i.e., types IIa, IIx, and IIb) and while diffuse atrophy has been reported here and in the literature [18], the subtle morphological and genetic differences between the mouse and rat transgenic models and the stage of disease progression may account for the discrepancies with the current work. Nevertheless, both studies confirm that HIV-1-related proteins have significant biological activity and induce systemic muscle atrophy.

We next identified the effect of HIV-1-related protein expression on oxidative stress and redox balance. Oxidative stress is a common complication in HIV-1-infected individuals and is likely responsible, at least in part, for cardiac and skeletal muscle myopathies [19]. Here, we show that both muscle types experience significant oxidative stress, with specific detriments to components of the GSH anti-oxidant cycle. Importantly, previous work has suggested that GSH replacement therapies using precursors such as L-glutamine in HIV-1-infected individuals successfully replenishes the available pool of GSH and preserves lean body mass [20]. Further, in combination with traditional highly active antiviral therapies (HAART), the adjunctive use of nutritional therapies like N-acetyl cysteine or α-lipoic acid supplementation may interrupt the process of viral activation and CD4 cell death [5,21]. Therefore, the inclusion of GSH replacement strategies in the treatment regimes of HIV-1-infected individuals may be warranted in order to reduce oxidative stress and possibly attenuate muscle catabolism. Based on our previous associations between alcohol-induced oxidative stress and atrogin-1 and TGFβ_1_expressions, GSH supplementation in HIV-1-infected individuals may have the added benefit of attenuating redox-sensitive mechanisms implicated in cardiac and skeletal muscle derangements [11].

Atrogin-1, also known as Muscle Atrophy F-box (MAFbx), and muscle ring finger protein-1 (MuRF-1) are E3 ubiquitin ligase that initiates ATP-dependent, ubiquitin-mediated proteolysis and are abundant in skeletal muscles undergoing atrophy [13,22]. However, the roles of these atrophy-related genes, or atrogenes [23], in the regulation of cardiac mass is more controversial. For example, atrogin-1 inhibited pathologic cardiac hypertrophy by initiating the degradation of calcineurin, a calcium-dependent phosphatase implicated in pathologic hypertrophy [24]. Further, both genes were decreased in unloading-induced cardiac atrophy [25]. In contrast, atrogin-1 mRNA levels were increased in hypertrophied rat hearts [26]. Here, both muscle types showed increased mRNA levels of atrogin-1 suggesting that this ubiquitin ligase plays an important role in regulating these defects. In support of this notion, skeletal muscles from cachectic, HIV-1-infected individuals showed a dramatic increase in the gene levels of 2.4 and 1.2 kb ubiquitin, and the C8 proteasome [27].

A recent report suggested that atrogin-1 may regulate TGFβ signaling by degrading specific substrates associated with this pathway [28]. TGFβ is a superfamily of pluripotent cytokines implicated in skeletal muscle catabolic conditions and in the development of cardiac fibrosis [29,30]. Interstitial and myocardial fibrosis has been reported in HIV-infected patients [31,32], and while we did not directly test for the presence of myocardial fibrosis, gene levels of the pro-fibrotic cytokine TGFβ_1 _were significantly upregulated in the hearts of transgenic rats. Further, in light of the evolving evidence implicating atrogin-1 and TGFβ_1 _in the pathophysiology of these muscle derangements, our findings suggest a mechanistic relationship between HIV-1-induced oxidative stress and these catabolic mechanisms. Taken together, our data support the hypothesis that these redox-sensitive inductions of catabolic factors by HIV-1-related proteins represent significant clinical alterations in the evolution of HIV-1 myopathies that are responsible, at least in part, for the establishment of a catabolic signaling milieu.

## Conclusion

Using a unique HIV-1 transgenic rat model, we provide compelling experimental evidence that HIV-1-related protein expression, in the absence of viral replication, is sufficient to reproduce many clinical manifestations commonly described in the human condition, including increased heart mass, skeletal muscle atrophy and oxidative stress. These muscle derangements may be due in part to specific alterations in redox-sensitive thiols including cysteine and glutathione. We also determined that heart and plantaris muscles from HIV-1 transgenic rats have increased levels of the redox-sensitive catabolic factors. Therefore, if this pathophysiological scheme identified in this HIV-1 transgenic model proves to be relevant to the human condition, this study suggests that dietary supplementation with cysteine or other glutathione precursors could modulate oxidative stress and/or redox-sensitive signaling events and decrease skeletal and cardiac myopathy in HIV-1-infected individuals.

## Methods

### Animals and tissue collections

Male, Fischer 344/NHsd HIV-1 transgenic rats (hemizygous NL4-3Δgag/pol) [14] and wild type Fischer 344/NHsd rats (~400 g, n = 6/group) were purchased from Harlan (Indianapolis, Indiana) and housed in pairs under a 12:12 light-dark cycle. Animals had free access to food and water. All procedures were approved by Atlanta Veteran Affairs Medical Center Institutional Animal Care and Use Committee.

Rats were anesthetized with sodium pentobarbital, heart and plantaris muscles were removed, blotted dry, weighed and prepared for further analyses. For measures involving heart tissue, ventricles were separated from atria and used for all experiments.

### Plantaris morphology & MHC isoform expression

Plantaris muscles were embedded in OCT and immediately frozen in isopentane cooled in liquid nitrogen. Serial sections from the mid-belly of the plantaris muscle were cut at 14 or 8 μm for analyses of CSA or MHC isoform determination, respectively. All incubations were performed at room temperature. For CSA determination, plantaris sections were adhered to superfrost slides, processed for hematoxylin and eosin staining, dehydrated and mounted. For MHC isoform determination, sections were processed for immunohistochemical detection of slow or fast MHC protein expression using the ABC method (Vector Labs, Burlingame, California). Sections were rehydrated in phosphate buffered saline (PBS, pH 7.4), incubated in blocking solution for 20 min, and then incubated in anti-slow MHC or anti-fast MHC IgG (Sigma, St. Louis, Missouri) for 90 min. Sections were washed in PBS, incubated in biotinylated secondary antibody for 60 min, washed again in PBS, and then incubated in an avidin-rich solution for 60 min. After a final wash, positive biotin-avidin binding was observed with diaminobenzidine. All sections were visualized with a Leica microscope and measured using ImageJ software (NIH, Bethesda, Maryland). Approximately 125 fibers per muscle were analyzed. Data are expressed as the percentage of slow (type I), hybrid (co-expression of types I and II), and fast (type II) MHC types relative to the total pool of MHC isoforms.

### High performance liquid chromatography

For determining the levels of GSH, GSSG, Cys, and Cyss in heart and plantaris muscle tissues, we used a variation of the high performance liquid chromatography (HPLC) method previously described [11]. Briefly, each sample was extracted in 5% perchloric acid with 0.2 M boric acid and 10 μM γ-glutamyl-glutamate as an internal standard. Iodoacetic acid was added and the pH was adjusted to 9.0 ± 0.2. After incubation for 20 min to obtain S-carboxymethyl derivatives of thiols, dansyl chloride was added and the samples were incubated for 24 h in the dark. Samples were then separated on an amine column with solvents previously described [11]. Fluorescence detection was used for separation and quantification of the dansyl derivatives. The redox pairs (i.e., GSH and GSSG, Cys and Cyss) were measured in parallel and expressed as picomoles per milligram of plantaris tissue.

### Real-time polymerase chain reaction (RT-PCR)

Heart and plantaris samples were immediately frozen in liquid nitrogen and stored at -80°C until processed for RT-PCR analyses. Trizol was added (1 ml/100 mg tissue) and the tissues homogenized using an electric tissue homogenizer. Total RNA (2.5 μg) was reverse transcribed in a 40 μl final reaction volume using random primers and M-MLV reverse transcriptase (Invitrogen, Carlsbad, California). The reverse transcription reaction was incubated at 65°C for 10 min, 80°C for 3 min, and 42°C for 60 min. RT-PCR products were analyzed using the iCycler iQ system (Biorad, Hercules, California). cDNA (5 μl of a 1:10 dilution) was amplified in a 25 μl reaction containing 400-nm gene-specific primer pair and iQ Sybr Green Supermix (Biorad). Primers were as follows: atrogin-1, 5'-TCCAGACCCTCTACACATCCTT-3' and 5'-CCTCTGCATGATGTTCAGTTGT-3'; MuRF-1, 5'-ATCACTCAGGAGCAGGAGGA-3' and 5'-CTTGGCACTCAAGAGGAAGG-3'; TGFβ1, 5'-CTACTACGCCAAAGAAGTCACC-3' and 5'-CTGTATTCCGTCTCCTTGGTT-3'. Samples were incubated at 95°C for 15 min, followed by 40 cycles of denaturation, annealing, and extension at 95°C, 60°C, and 72°C, respectively. As a control, RT-PCR was also performed on 2 μl of each RNA sample to confirm absence of contaminating genomic DNA. Fluorescence was recorded at the end of each annealing and extension step. All reactions were performed in triplicate and the starting quantity of the gene of interest was normalized to 18S rRNA for each sample. The delta-delta Ct method was used to analyze alterations in gene expression and values were expressed as fold changes relative to control [11].

### Statistics

Student's t-tests were performed to analyze differences between HIV-1 transgenic and control rats. Significance was accepted at p ≤ 0.05.

## Abbreviations

CSA: cross-sectional area; Cys: cysteine; Cyss: cystine; GSH: glutathione; GSSG: glutathione disulfide; MAFbx: muscle atrophy F box (atrogin-1); MuRF-1: muscle ring finger protein-1; MHC: myosin heavy chain; TGFβ_1_: Transforming Growth Factor-β1.

## Competing interests

The authors declare that they have no competing interests.

## Authors' contributions

JSO: conception and design, data collection and analysis in cardiac and skeletal muscle tissues, figure and manuscript preparation. YIA: real time PCR analyses, contribution of important intellectual content. LAB: HPLC analyses of glutathione metabolites in cardiac and skeletal muscle tissues. DMG: design, editorial support and contribution of important intellectual content, research fund collection. All authors have approved of this final manuscript.

## References

[B1] Lewis W (2000). Cardiomyopathy in AIDS: A pathophysiological perspective. Prog Cardiovasc Dis.

[B2] Barbaro G (2003). Pathogenesis of HIV-associated heart disease. AIDS.

[B3] Gonzalez-Cadavid NF, Taylor WE, Yarasheski K, Sinha-Hikim I, Ma K, Ezzat S, Shen R, Lalani R, Asa S, Mamita M, Nair G, Arver S, Bhasin S (1998). Organization of the human myostatin gene and expression in healthy men and HIV-infected men with muscle wasting. Proc Natl Acad Sci USA.

[B4] Hack V, Schmid D, Breitkreutz R, Stahl-Henning C, Drings P, Kinsherf R, Taut F, Holm E, Droge W (1997). Cystine levels, cystine flux, and protein catabolism in cancer cachexia, HIV/SIV infection, and senescence. FASEB J.

[B5] Patrick L (2000). Nutrients and HIV: part three – N-acetylcysteine, alpha-lipoic acid, L-glutamine, and L-carnitine. Altern Med Rev.

[B6] Yarasheski KE, Smith SR, Powderly WG (2005). Reducing plasma HIV RNA improves muscle amino acid metabolism. Am J Physiol Endocrinol Metab.

[B7] Grody WW, Cheng L, Lewis W (1990). Infection of the heart by the human immunodeficiency virus. Am J Cardiol.

[B8] Raidel SM, Haase C, Jansen NR, Russ RB, Sutliff RL, Velsor LW, Day BJ, Hoit BD, Samarel AM, Lewis W (2002). Targeted myocardial transgenic expression of HIV Tat causes cardiomyopathy and mitochondrial damage. Am J Physiol Heart Circ Physiol.

[B9] Kan H, Xie Z, Finkel MS (2006). iPLA2 inhibitor blocks negative inotropic effect of HIV gp120 on cardiac myocytes. J Mol Cell Cardiol.

[B10] Kan H, Xie Z, Finkel MS (2004). p38 MAP kinase-mediated negative inotropic effect of HIV gp120 on cardiac myocytes. Am J Physiol Cell Physiol.

[B11] Otis JS, Brown LA, Guidot DM (2007). Oxidant-induced atrogin-1 and transforming growth factor-beta1 precede alcohol-related myopathy in rats. Muscle Nerve.

[B12] Chariot P, Dubreuil-Lemaire ML, Zhou JY, Lamia B, Dumé L, Larcher B, Monnet I, Levy Y, Astier A, Gherardi R (1997). Muscle involvement in human immunodeficiency virus-infected patients is associated with marked selenium deficiency. Muscle Nerve.

[B13] Bodine SC, Latres E, Baumhueter S, Lai VK, Nunez L, Clarke BA, Poueymirou WT, Panaro FJ, Na E, Dharmarajan K, Pan ZQ, Valenzuela DM, DeChiara TM, Stitt TN, Yancopoulos GD, Glass DJ (2001). Identification of ubiquitin ligases required for skeletal muscle atrophy. Science.

[B14] Reid W, Sadowska M, Denaro F, Rao S, Foulke J, Hayes N, Jones O, Doodnauth D, Davis H, Sill A, O'Driscoll P, Huso D, Fouts T, Lewis G, Hill M, Kamin-Lewis R, Wei C, Ray P, Gallo RC, Reitz M, Bryant J (2001). An HIV-1 transgenic rat that develops HIV-related pathology and immunologic dysfunction. Proc Natl Acad Sci USA.

[B15] Reid W, Abdelwahab S, Sadowska M, Huso D, Neal A, Ahearn A, Bryant J, Gallo RC, Lewis GK, Reitz M (2004). HIV-1 transgenic rats develop T cell abnormalities. Virology.

[B16] Lipshultz SE, Easley KA, Orav EJ, Kaplan S, Starc TJ, Bricker JT, Lai WW, Moodie DS, McIntosh K, Schluchter MD, Colan SD (1998). Left ventricular structure and function in children infected with human immunodeficiency virus: the prospective P2C2 HIV Multicenter Study. Circulation.

[B17] Serrano AL, Jardi M, Suelves M, Klotman PE, Munoz-Canoves P (2008). HIV-1 transgenic expression in mice induces selective atrophy of fast-glycolytic skeletal muscle fibers. Front Biosci.

[B18] Mhiri C, Bélec L, Di Costanzo B, Georges A, Gherardi R (1992). The slim disease in African patients with AIDS. Trans R Soc Trop Med Hyg.

[B19] Stehbens WE (2004). Oxidative stress in viral hepatitis and AIDS. Exp Mol Pathol.

[B20] Shabert JK, Winslow C, Lacey JM, Wilmore DW (1999). Glutamine-antioxidant supplementation increases body cell mass in AIDS patients with weight loss: a randomized, double-blind controlled trial. Nutrition.

[B21] Droge W, Holm E (1997). Role of cysteine and glutathione in HIV infection and other diseases associated with muscle wasting and immunological dysfunction. FASEB J.

[B22] Gomes MD, Lecker SH, Jagoe RT, Navon A, Goldberg AL (2001). Atrogin-1, a muscle-specific F-box protein highly expressed during muscle atrophy. Proc Natl Acad Sci USA.

[B23] Sacheck JM, Ohtsuka A, McLary SC, Goldberg AL (2004). IGF-I stimulates muscle growth by suppressing protein breakdown and expression of atrophy-related ubiquitin ligases, atrogin-1 and MuRF1. Am J Physiol Endocrinol Metab.

[B24] Li HH, Willis MS, Lockyer P, Miller N, McDonough H, Glass DJ, Patterson C (2007). Atrogin-1 inhibits Akt-dependent cardiac hypertrophy in mice via ubiquitin-dependent coactivation of Forkhead proteins. J Clin Invest.

[B25] Sharma S, Ying J, Razeghi P, Stepkowski S, Taegtmeyer H (2006). Atrophic remodeling of the transplanted rat heart. Cardiology.

[B26] Razeghi P, Baskin KK, Sharma S, Young ME, Stepkowski S, Essop MF, Taegtmeyer H (2006). Atrophy, hypertrophy, and hypoxemia induce transcriptional regulators of the ubiquitin proteasome system in the rat heart. Biochem Biophys Res Commun.

[B27] Llovera M, Garcia-Martinez C, Agell N, Lopez-Soriano FJ, Authier FJ, Gherardi RK, Argiles JM (1998). Ubiquitin and proteasome gene expression is increased in skeletal muscle of slim AIDS patients. Int J Mol Med.

[B28] Aoyama Y, Urushiyama S, Yamada M, Kato C, Ide H, Higuchi S, Akiyama T, Shibuya H (2004). MFB-1, an F-box-type ubiquitin ligase, regulates TGF-beta signaling. Genes Cells.

[B29] Lim H, Zhu YZ (2006). Role of transforming growth factor-beta in the progression of heart failure. Cell Mol Life Sci.

[B30] Lundberg IE (2000). The role of cytokines, chemokines, and adhesion molecules in the pathogenesis of idiopathic inflammatory myopathies. Curr Rheumatol Rep.

[B31] De Castro S, d'Amati G, Gallo P, Cartoni D, Santopadre P, Vullo V, Cirelli A, Migliau G (1994). Frequency of development of acute global left ventricular dysfunction in human immunodeficiency virus infection. J Am Coll Cardiol.

[B32] Lanjewar DN, Katdare GA, Jain PP, Hira SK (1998). Pathology of the heart in acquired immunodeficiency syndrome. Indian Heart J.

